# Ecological shifts in soil microbiota and root rot disease progress during ginseng monoculture

**DOI:** 10.3389/fmicb.2024.1442208

**Published:** 2024-10-18

**Authors:** Gyeongjun Cho, Da-Ran Kim, Youn-Sig Kwak

**Affiliations:** ^1^Division of Agricultural Microbiology, National Institute of Agriculture Science, Rural Development Administration, Wanju, Republic of Korea; ^2^Division of Applied Life Science, RILS, Gyeongsang National University, Jinju, Republic of Korea

**Keywords:** influencer taxa, ginseng, keystone taxa, root rot disease, suppressive soil

## Abstract

**Introduction:**

The phenomenon in which the damage of plant diseases is suppressed by continuous cropping is defined as “suppressiveness” and the development of suppressive soils and key beneficial microorganisms have been identified through various previous studies. However, no studies have been conducted on microbial communities related to disease occurrence before the initial occurrence of diseases in crop monoculture.

**Methods:**

We aimed to investigate the ecological modifications of pathogen population density in soil, disease occurrence rate, and microbiota community shifting during ginseng monoculture to better understand the tripartite social relationships in the monoculture system. To achieve the study’s objectives, a long-term monoculture of ginseng was established. The microbial diversity and community structure were analyzed using high-throughput sequencing, and the pathogen population density and disease occurrence rate were determined using qPCR and observation.

**Results and discussion:**

The results showed that the initial rhizosphere bacterial community of ginseng had already collapsed before the development of the root rot disease. The study also identified the crucial role of soil-borne pathogens in causing disease and the loss of initial keystone taxa populations in the early stages of monoculture. Our study revealed a novel aspect of soil microbiota dynamics during ginseng monoculture, with seven distinct microbes (Beijerinckiaceae, Comamonadaceae, Devosiaceae, Rhizobiaceae, Sphingobacteriaceae, Sphingomonadaceae, and Xanthomonadaceae) participating in soil nitrogen metabolism as an ‘initial community’ that regulates root rot disease through nutritional competition. The findings contribute to ecological research on disease-suppressiveness soil, disease management, and sustainable agriculture.

## Introduction

Ginseng (*Panax ginseng* C. A. Meyer, Araliaceae) has been traditionally used as a medicinal herb in Northeast Asia, including Korea, China, and Japan, for over 2,000 years. Modern research has documented its potential anticancer properties, neuroprotective effect, heart-protective effect, and anti-inflammatory activity (Wong et al., 2015; Wei, 2016; Zheng et al., 2012; Saba et al., 2018). The active ingredients of ginseng are ginsenosides, which are steroidal saponins, protopanaxadiols, and protopanaxatriols (Ratan et al., 2021). For both edible and therapeutic products, ginseng should be cultivated in one place for 4–6 years, due to its slow growth rate. However, this extended cultivation period makes it susceptible to soil-borne diseases, leading to continuous cultivation difficulties in one field (Park, 2001). In Korea, a major global producer of ginseng, it has been reported that disease results in a yield loss of 30–60% (Kim et al., 2012). The major soil-borne pathogens causing root rot are *Cylindrocarpon destructans* and *Fusarium* spp. (Park, 2001). The dominant species in the *Fusarium* genus include *F. solani, F. oxysporum*, and *F. moniliforme* (Lee, 2004). The temperature range for the growth of *C. destructans* has been reported to be 18°C for a highly virulent strain and 20°C for a low virulent strain (Rahman and Punja, 2005). On the other hand, the optimal growth temperature for *F. solani*, a major root rot pathogen within the *Fusarium* spp., has been documented to be 30°C with high pathogenicity observed at 25–30°C (Yan and Nelson, 2020). In recent studies of the fungal community in *Panax ginseng*, *Fusarium* has been found to be more prevalent than *Cylindrocarpon*, possibly due to the impact of global warming (Wong et al., 2015). The pathogenicity of the *Fusarium* spp. is closely linked to the diverse mycotoxins they produce (Urbaniak et al., 2020).

Plants and their environment form a specific phytobiome, which refers to the plant’s ecological area, including the plant’s biological interactions with both living and non-living objects in its vicinity. This term encompasses the rhizosphere, a narrow region of soil influenced by root exudates and inhabited by specific microbes, shaped by the plant phenotype (Leach et al., 2017). The microbial community in the rhizosphere plays a crucial role in plant health and productivity, with examples such as suppressive soil, which prevents plant diseases through the presence of beneficial microbes (Philippot et al., 2013; Weller et al., 2002). In contrast, the endosphere, intracellular regions within plant tissues, was once believed to be devoid of microorganisms in healthy plants, dating back to the 19th century (Company et al., 2012). However, this assumption was challenged by the first report and hypothesis of microbe isolation from the endosphere by Galippe (1887). Currently, it is widely accepted that various plant organs host diverse endophytic bacteria and fungi, which contribute to plant development, nutrient acquisition, disease suppression, and tolerance to abiotic stress (White et al., 2019). The microbiota that colonizes both the rhizosphere and endosphere interact intricately with one another, strongly influencing the host plant (Vandenkoornhuyse et al., 2015).

In agroecosystems, most crops lack resistance genes against necrotrophic pathogens, which contributes to the exacerbation of soil diseases in monoculture systems. Nonetheless, it has been observed that disease outbreaks in crop monocultures tend to spontaneously decline after a specific occurrence of severe disease (Weller and Cook, 1983). The natural suppression of take-all disease in wheat monocultures has been attributed to the presence of *Pseudomonas* in the rhizosphere (Weller and Cook, 1983; Kwak and Weller, 2013). Similarly, *Streptomyces*, which is known to inhibit the development of Fusarium wilt, a major contributor to crop damage in strawberry cultivation, achieves this through the secretion of various lantipeptide antibiotics from the strawberry rhizosphere (Cha et al., 2016; Kim et al., 2019a; Kim et al., 2019b). Previous studies on disease suppressiveness have focused on the discovery and characterization of microbial agents that contribute to the suppression of disease in suppressive soils.

This study aims to examine the changes in the microbiota community structure and the collapse of the early microbial community involved in soil integrity during the “conducive soil” period when the initiation of diseases is at its maximum in the early stages of monoculture. To achieve these objectives, a monoculture model system of ginseng was established. The investigation focused on the shift in the microbiota community and the identification of early keystone taxa.

## Materials and methods

### Ginseng cultivation

Pebbles (<10 cm) were placed at the bottom of the pot (round: 15 cm diameter × 20 cm high) and approximately 1 kg of soil was added. The soil mixture consisted of 50% autoclaved sand, 40% autoclaved ginseng field soil, and 10% raw ginseng field soil to inoculate native microbiota. The raw ginseng field soil, located at 36°56′34.9” N: 127°44′59.7″ E, had been used for the monoculture of ginseng for 9 years. Twenty-five ginseng seedlings (1 year old) were planted in each pot and grown with 3-day intervals for watering and no fertilization. The ginsengs were cultured under light (25°C for 16 h) and dark (20°C for 8 h) conditions for 20 days to allow for sampling and phenotypic measurements. After the study, new leafless ginseng seedlings were replanted in the used soil as an artificial monoculture condition. This process was repeated 10 times (Supplementary Figure S1).

### Phenotype and disease recording

The cultured ginsengs were evaluated based on new leaf sprout rate, stem length, and root weight. The progression of root rot disease was also recorded, and each ginseng was scored according to the extent of the disease symptoms. Disease index score (DIS) value was followed: a score of 0 was assigned for no damage, 1 for damage to only fine roots, five for damage <10%, 30 for damage between 10 and 50%, and 75 for damage between 50 and 100% (Cho et al., 2023). The average DIS was calculated for each pot unit (Supplementary Figure S2). Phenotype recording and DIS according to the monoculture of ginseng were performed with 10 biological replications for each cycle.

### Rhizosphere and endosphere microbiota collection

The ginseng roots were first stripped of their stems and then gently shaken to remove any soil. The rhizosphere was then separated from the roots through a 20-min sonication process (at 4°C)in 200 mL of phosphate buffered saline (PBS) with a pH of 7.4. The separated rhizosphere microbiota was collected by centrifugation at 3,000× g for 20 min using a centrifuge (LaboGene, Seoul, Republic of Korea). The collected sample was stored at −80°C. The endophyte collection process was a modified version of the nycodenz density gradient centrifugation method (Ikeda et al., 2009). The rhizosphere-separated roots were washed twice with autoclaved distilled water after being disinfected with 3% NaOCl for 3 min and 70% ethanol for another 3 min. Approximately 20 to 30 roots were blended with 100 mL of bacterial cell extraction buffer (BCE) (1% Triton X-100, 50 mM Tris–HCl pH7.5; 2 mM 2-mercaptoethanol) for 1 min at 18,000 × g using a Ninja^®^ BL450 blender (SharkNinja, Needham, MA, USA). The blended mixtures were filtered through a sterilized Miracloth (Merck Millipore, Burlington, MA, USA) into a 50 mL conical tube. The samples were then centrifuged at 500 × g for 10 min at 10°C, and the resulting supernatant was transferred to a new tube. Further centrifugation was performed at 5,500 × g for 20 min at 10°C, and the resulting pellet was discarded, and 10 mL of BCE buffer was added to the supernatant. The sample was subjected to two additional rounds of centrifugation at 10,000 × g for 10 min at 10°C, with the supernatant discarded and the pellet resuspended in 10 mL of BCE buffer each time. The mixture was then washed once more with 10 mL of BCE buffer. The resulting pellet, resuspended in 5 mL of 50 mM Tris–HCl (pH 7.5), was carefully overlayed on a 3 mL solution of Nycodenz™ (AXIS-SHIELD PoC AS, Oslo, Norway) (8 g Nycodenz™ dissolved in 10 mL of 50 mM Tris–HCl pH 7.5) in a 15 mL conical tube. The tube was centrifuged at 12,000 x g for 90 min at 10°C. The white layer, composed primarily of bacterial cells, was transferred to a 1.5 mL tube and stored at −80°C after being replaced with 20% glycerol dissolved in PBS.

### Microbial total DNA extraction

DNA extraction from the rhizosphere was performed using the FastDNA™ SPIN Kit for Soil (MP Biomedicals, Santa Ana, CA, USA). The rhizosphere samples were homogenized in a Lysing Matrix E tube with 978 μL sodium phosphate buffer and 122 μL MT buffer. The homogenization was carried out using the FastPrep-24™ Classic Instrument (MP Biomedicals) at a speed of 6.0 for 40 s. After centrifugation at 18,000 × g for 10 min, 250 μL of protein precipitation solution was added to the supernatant and transferred to a clean 2 mL tube. The solution was then inverted 10 times and subjected to another round of centrifugation. The transferred supernatant was added to 1 mL of resuspended Binding Matrix in a 15 mL conical tube and inverted for 2 min. The matrix was then allowed to settle for 3 min, after which the top 500 μL of the supernatant was discarded without losing any matrix. The matrix in the supernatant was collected using the SPIN™ filter and washed with 500 μL SEWS-M using centrifugation. Finally, the rhizosphere DNA bound to the matrix was extracted using 50 μL of DNase-free water. The endophyte DNA extraction protocol utilized in this study was based on the method described by Goldenberger et al. (1995). Endophytic bacterial cells were resuspended in 500 μL of lysis buffer containing 10 mM Tris–HCl (pH 8.5), 1 mM EDTA, 0.5% SDS, and 200 μg/mL of proteinase K. The sample was then incubated at 55°C for 3 h and 95°C for 10 min. Finally, the sample was centrifuged at 4°C for 10 min.

### Quantitative polymerase chain reaction (qPCR)

The density of *F. solani* cells in the rhizosphere was determined using quantitative polymerase chain reaction (qPCR). The SYBR^®^ Green Realtime PCR Master Mix (Toyobo, Osaka, Japan) and CFX™ Connect (Bio-Rad, Hercules, CA, USA) were used for the analysis. The forward and reverse primers used in the experiment were ITS1F (5’-CTTGGTCATTTAGAGGAAGTAA-3′) and AFP346 (5’-GGTATGTTCACAGGGTTGATG-3′) (Lievens et al., 2006). To convert the results to colony-forming units (CFUs), a sample of 10^7^ CFU/gram of soil concentration derived from sand stock was subjected to 1/10 serial dilution The results of qPCR and the 1/10 serially diluted CFU/soil g values were used to create a standard curve. This standard curve was then used to measure the density of the pathogen from rhizosphere DNA. The qPCR thermal conditions were optimized to start with 1 min at 95°C, followed by 45 cycles of 15 s at 95°C, 15 s at 60°C, and 30 s at 72°C. At the end of each cycle, and during the melt curve analysis, the fluorescence was measured by reheating to 95°C and cooling to 60°C, with excitation at 497 nm and emission at 520 nm.

### 16S rRNA V4 library sequencing

Rhizosphere and endophyte DNA were sequenced by consigning to Macrogen Inc. (Seoul, Republic of Korea) using the MiSeq 2 × 300 bp platform (Illumina, San Diego, CA, USA). To eliminate chloroplast and mitochondria sequences, the endosphere DNA was subjected to polymerase chain reaction (PCR) treatment using peptide nucleic acid (PNA) clamps (0.75 μM mPNA: 5’-GGCAAGTGTTCTTCGGA-3′ and 0.75 μM pPNA: 5’-GGCTCAACCCTGGACAG-3′) with the aid of KAPA HiFi HotStart ReadyMix (Kapa Biosystems, Wilmington, MA, USA) (Lundberg et al., 2013). This process was performed prior to consignment. The PNA-mediated PCR condition was established using two primers at a concentration of 0.4 μM each, 27F (5’-AGAGTTTGATCCTGGCTCAG-3′) and 1492R (GGTTACCTTGTTACGACTT). The thermal cycling protocol consisted of pre-denaturation at 98°C for 3 min, followed by 25 cycles of denaturation at 98°C for 10 s, PNA annealing at 78°C for 10 s, primer annealing at 55°C for 30 s, extension at 72°C for 1 min, and a final extension step at 72°C for 5 min. The Illumina adapter-tagged primers used were 515F (5’-TCGTCGGCAGCGTCAGATGTGTATAAGAGACAGGTGCCAGCMGCCGCGGTAA-3′) and 806R (5’-GTCTCGTGGGCTCGGAGATGTGTATAAGAGACAGGGACTACHVHHHTWTCTAAT-3′). The first PCR thermal condition consisted of initial denaturation at 95°C for 3 min, followed by 25 cycles of denaturation at 95°C, primer annealing at 55°C, and extension at 72°C, each step lasting for 30 s, and final extension at 72°C for 5 min. The second PCR was performed to attach the linker and barcode sequences produced by Illumina, with the thermal cycling protocol consisting of initial denaturation at 95°C for 3 min, followed by eight cycles of denaturation at 95°C, primer annealing at 55°C, and extension at 72°C, each step lasting for 30 s, and final extension at 72°C for 5 min.

### *In silico* analysis

In this study, a used workstation was configured with Ubuntu 18.04 and equipped with 64 GiB DDR4 RAM and an AMD Ryzen Threadripper 3,970X processor. The MiSeq results were received in FASTQ format and underwent quality control using the DADA2 (version 1.16.0) package in R (version 4.0.3). The reads were trimmed by removing the 5′-end corresponding to 515F (19 bp) and 806R (20 bp) and removing the 3′-end with an average quality score of 30 points or less. The error rate was machine-learned, the sequences were corrected, the forward and reverse reads were merged, and the equal reads were clustered using the divisive amplicon denoising algorithm (DADA) (Callahan et al., 2016). The amplicon sequence variants (ASVs) were then assigned by the IDTAXA algorithm (version 2.16.1) (Murali et al., 2018), which was found to be less over-classified compared to the naive Bayesian classifier, using the DECIPHER (version 2.16.1) (Wright, 2016) and SILVA databases. After removing the chimeric ASVs, the change in the number of reads was recorded and is shown in Supplementary Table S1. The reads were not normalized based on the minimum reads sample, as it already shows sufficient convergence on the rarefaction curve (Supplementary Figure S3). Additionally, when these reads are converted to relative abundance for analysis, the normalization does not present a significant issue.

The evaluation of diversity and community construction was performed using R packages, including vegan (version 2.5–0),^1^ phyloseq (version 1.32.0),^2^ and others, as well as FastSpar (version 0.0.10) (Watts et al., 2019),^3^ which is a C++ implementation of the SparCC algorithm. The distinction between stochastic community turnover was made using the *β*-nearest taxon index (βNTI) and Raup-Crick dissimilarity (βRC). The calculation of βNTI was performed using the aligned sequence and phylogenetic tree, which were computed using the maximum likelihood TN93 + G4 model in the endosphere and the HKY85 + G4 model in the rhizosphere, both obtained from DECIPHER. The aligned sequence and phylogenetic tree calculated were also obtained from DECIPHER. The function of the bacterial community was predicted using the phylogenetic investigation of communities by reconstruction of unobserved states (PICRUSt2; version 2.3.0-b) (Douglas et al., 2020). The predictions were based on the conversion of 16S rRNA amplicon sequencing results to whole metagenome shotgun sequencing results using Python (version 3.7.8). The statistical evaluation of the predictions was carried out using DESeq2 (version 1.28.1).^4^ The results were visualized using ggplot2 (version 3.3.2).^5^ The R code used in the study is available on the github repository.^6^

### Nitrogen fixation analysis with semi-solid Nfb media

A group of bacteria that were considered important for disease control were isolated or obtained from microbial banks (Table 1). The nitrogen fixation test was performed using nitrogen-free bromothymol blue malate broth (Nfb), which was prepared by dissolving the following ingredients in 1 liter of water: 5 g malic acid, 0.5 g K_2_HPO_4_, MgSO_4_·7H_2_O, 0.1 g NaCl, 0.02 g CaCl_2_·2H_2_O, 4.5 g KOH, 0.080 mg CuSO_4_·5H_2_O, 0.024 mg ZnSO_4_·7H_2_O, 2.8 mg H_3_BO_3_, 2 mg NaMoO_4_· 2H_2_O, 2.35 mg MnSO_4_·H_2_O, 10 mg bromothymol blue, 65.6 mg Fe(III)-EDTA, 0.1 mg biotin, and 0.2 mg pyridoxal-HCl. The pH was adjusted to 6.5 and the mixture was supplemented with 0.5% agar. The nitrogen fixation ability of the bacteria was measured using a spectrophotometer, specifically by measuring the absorbance at 602 nm. The bromothymol blue changes from bright green to blue in response to changes in airborne nitrogen, which increases the pH. This color change is a reliable indicator of the bacteria’s nitrogen fixation ability. To measure the nitrogen fixation ability, the bacteria were incubated at 28°C for 2 weeks in semi-solid Nfb. A loop of bacteria in autoclaved water with an absorbance of 0.1 was inoculated into the semi-solid Nfb.

**Table 1:** Isolated and obtained strain information corresponding to the influencers in the network analysis.

Name	Isolated place	Distribution	Distribution from	16S rRNA V4 library		BLAST	
				ASV ID	Family	Identities (bp/bp)	Gaps (%)
*Bosea* sp. WR72a	Korean ginseng rhizosphere	○	Korean Agricultural Culture Collection	ASV 109	Beijerinckiaceae	253/253	0
*Variovorax ginsengisoli* Gsoil 3,165	Korean ginseng rhizosphere	○	Korean Collection for Type Cultures	ASV 33	Comamonadaceae	253/253	0
N7R2	Korean ginseng rhizosphere (In this study)	–	–	ASV 2	Xanthomonadaceae	253/253	0
*Olivibacter soli* Gsoil 034	Korean ginseng rhizosphere	○	Korean Collection for Type Cultures	ASV 97	Sphingobacteriaceae	253/253	0
N10R5	Korean ginseng rhizosphere (In this study)	–	–	ASV 14	Rhizobiaceae	253/253	0
*Devosia neptuniae* BO184	Plantation soil	○	National Institute of Biological Resources of Korea	ASV 50	Devosiaceae	253/253	0
*Sphingopyxis panaciterrae* Gsoil 124	Korean ginseng rhizosphere	○	Korean Collection for Type Cultures	ASV 67	Sphingomonadaceae	253/253	0

## Results

### *Fusarium solani* population density in soil effect on ginseng root rot severity

**Figure 1:**
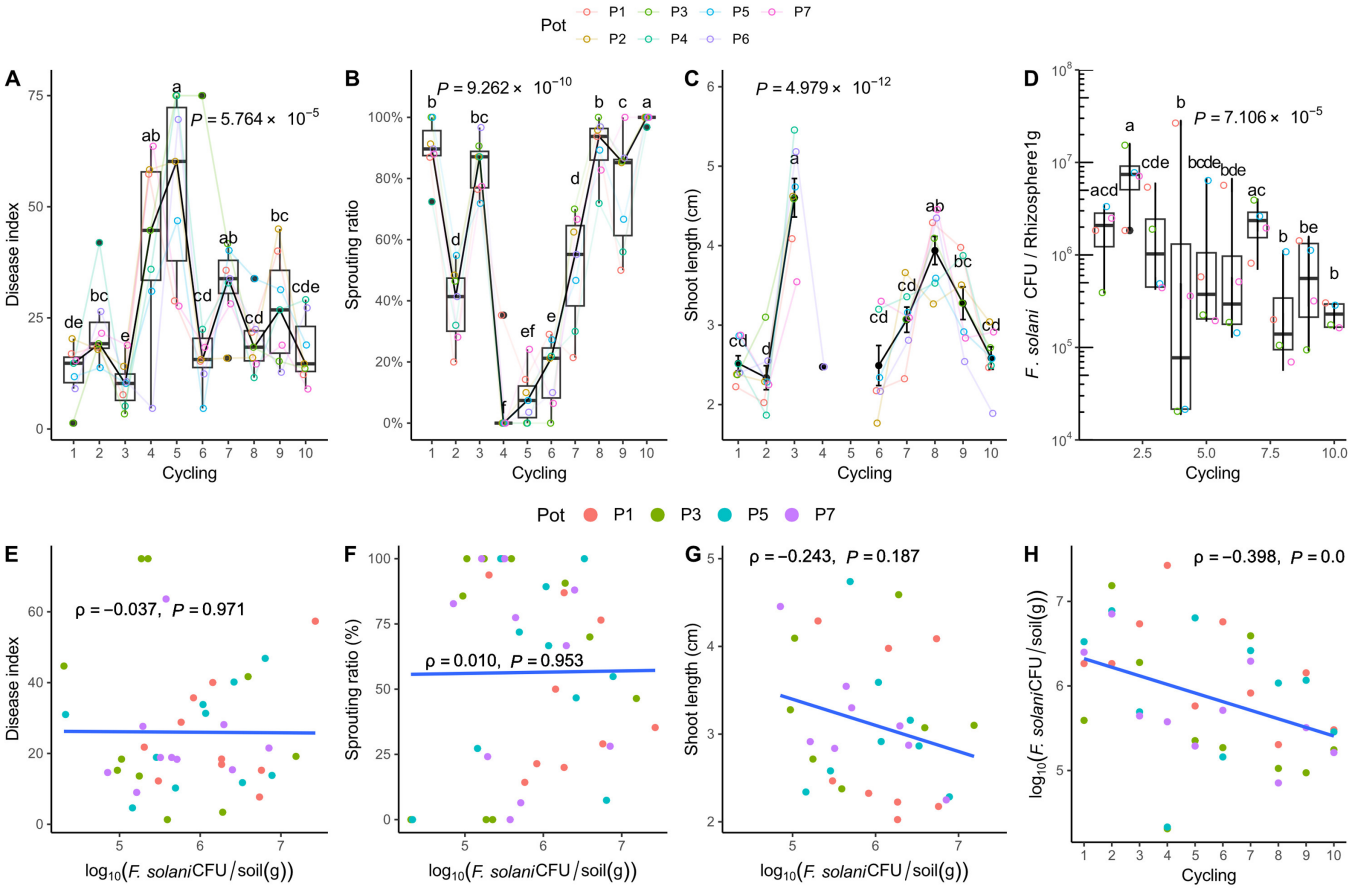
Phenotypic characteristics and *Fusarium solani* density in the rhizosphere. **(A)** Root rot disease index, **(B)** Sprouting ratio, **(C)** Shoot length, and **(D)** *F. solani* density. In the 5th cycle, severe root rot disease symptoms are observed, and the shoot length is not measured in the 4th and 5th cycling. Analysis of variance and Tukey’s test are conducted on the shoot length data after confirming normality and equal variance. The results are visualized by the black points representing the mean and the vertical bars representing the standard deviation. For the data without shoot length, the Kruskal-Wallis test is performed, and Conover’s test is used as a post-hoc analysis, depicted by the box-whisker plot. The *p* values in the plots represent the results of the Kruskal-Wallis test and analysis of variance. The significantly divided groups are indicated by letters on the whiskers and the mean points, based on the post-hoc tests (*p* < 0.05). Pearson correlations are conducted to assess the relationship between the *F. solani* density and growth phenotype. **(E)** Disease index, **(F)** sprouting ratio, **(G)** shoot length, and **(H)** plant cycling and *F. solani* density. A value of *ρ* = 0 indicates no relationship. Positive linear correlation is indicated as ρ approaches 1 and negative linear as ρ approaches −1. The results are statistically significant at *p* < 0.05. The *F. solani* density isfound to decrease significantly as the cycle progressed [*n* = 7 in **(A–C)**; *n* = 4 in **(D)**; *n* = 40 in **(E–H)**].

Root rot disease was the most progressed at the fifth cycle (Figure 1). Accordingly, a low sprouting ratio was observed at around the fifth cycle when the disease was the most severe, and there was no difference from the fourth to sixth cycle. To evaluate the effect of root rot occurrence with *F. solani*, cell density was measured by qPCR. Pearson analysis to determine linear correlation confirmed that there was no significant correlation between root rot or these phenotypes affected by the disease and the density of *F. solani* in soil. As the planting cycling progressed, the pathogen density decreased from 10^6^ to 10^5^ CFU/g of soil. However, the root rot disease occurrence showed no correlation with the pathogen population density (Figure 1).

### Rhizosphere network structure divided into two groups during monoculture

**Figure 2:**
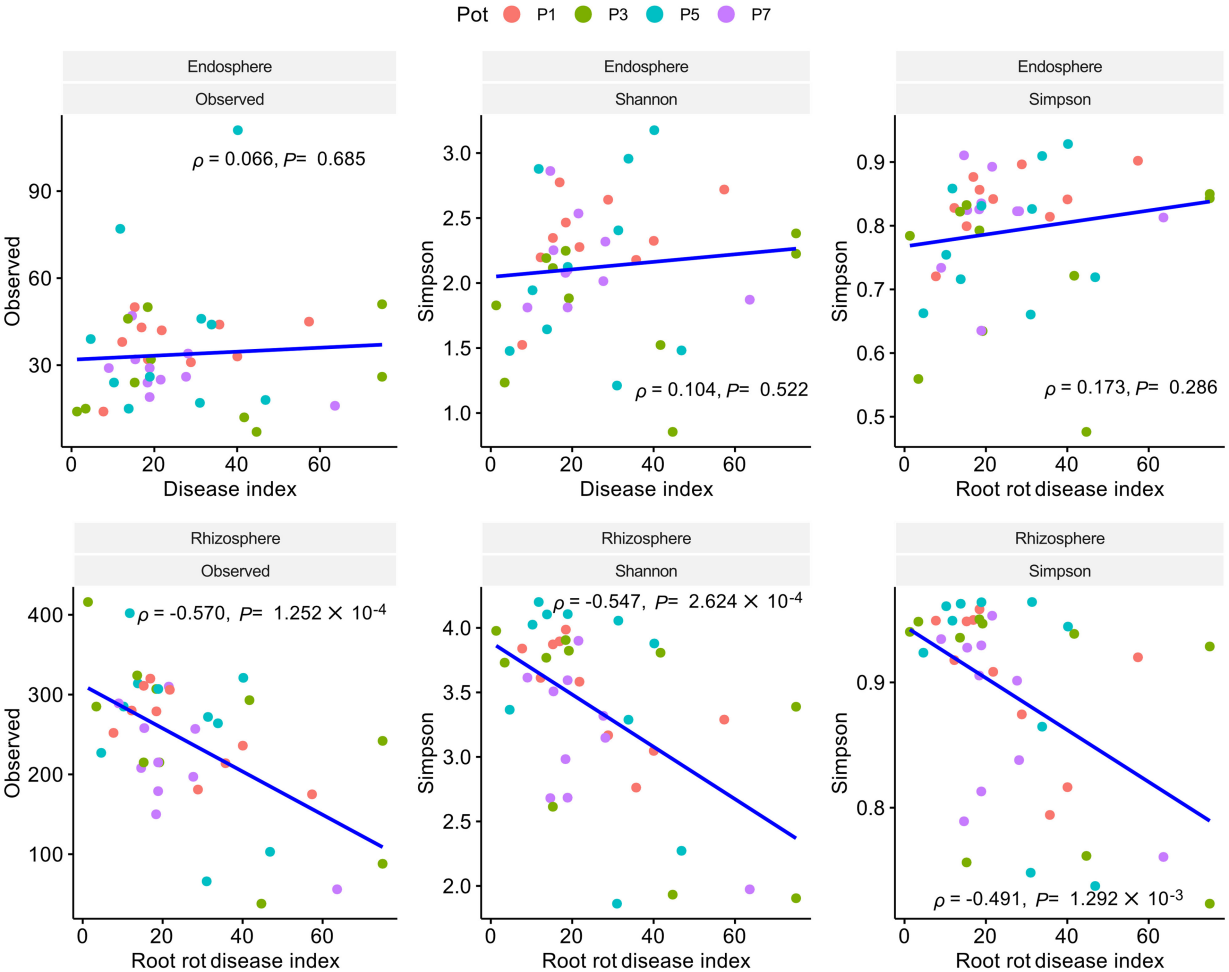
Pearson correlation analysis of *α* diversity and root rot disease index in the endosphere and rhizosphere. A statistical linear correlation is indicated by | ρ | approaching 1. Results reveal a negative correlation between rhizosphere alpha diversity and the disease index (*p* < 0.05, ρ < 0 with a negative correlation of −1 > ρ > 0). No correlation is observed between endosphere alpha diversity and the disease index.

To investigate the relationship between bacterial community and root rot progression, the bacterial 16S rRNA V4 region was sequenced using Illumina MiSeq. The obtained reads were deemed sufficient to represent the community, as indicated by the rarefaction curve, which showed that new ASVs were unlikely to be discovered as the sequencing continued (Supplementary Figure S3). The rhizosphere *α* diversity, which reflects the richness and evenness of ASVs, was found to be higher than that of the endosphere (Supplementary Figure S4A). In contrast, there was no correlation between endosphere α diversity and root rot, while a significant negative correlation was observed between rhizosphere α diversity and the root rot disease index (Figure 2). The top 10 families in the rhizosphere and endosphere were determined by calculating the average number of reads belonging to each family. These families included Pseudomonadaceae, Xanthomonadaceaea, Rhizobiaceae, Alcaligenaceae, Micrococcaceae, Microbacteriaceae, Comamonadaceae, Sphingobacteraceae, Staphylococaceae, and Flavobacteraceae (Supplementary Figure S4B).

**Figure 3:**
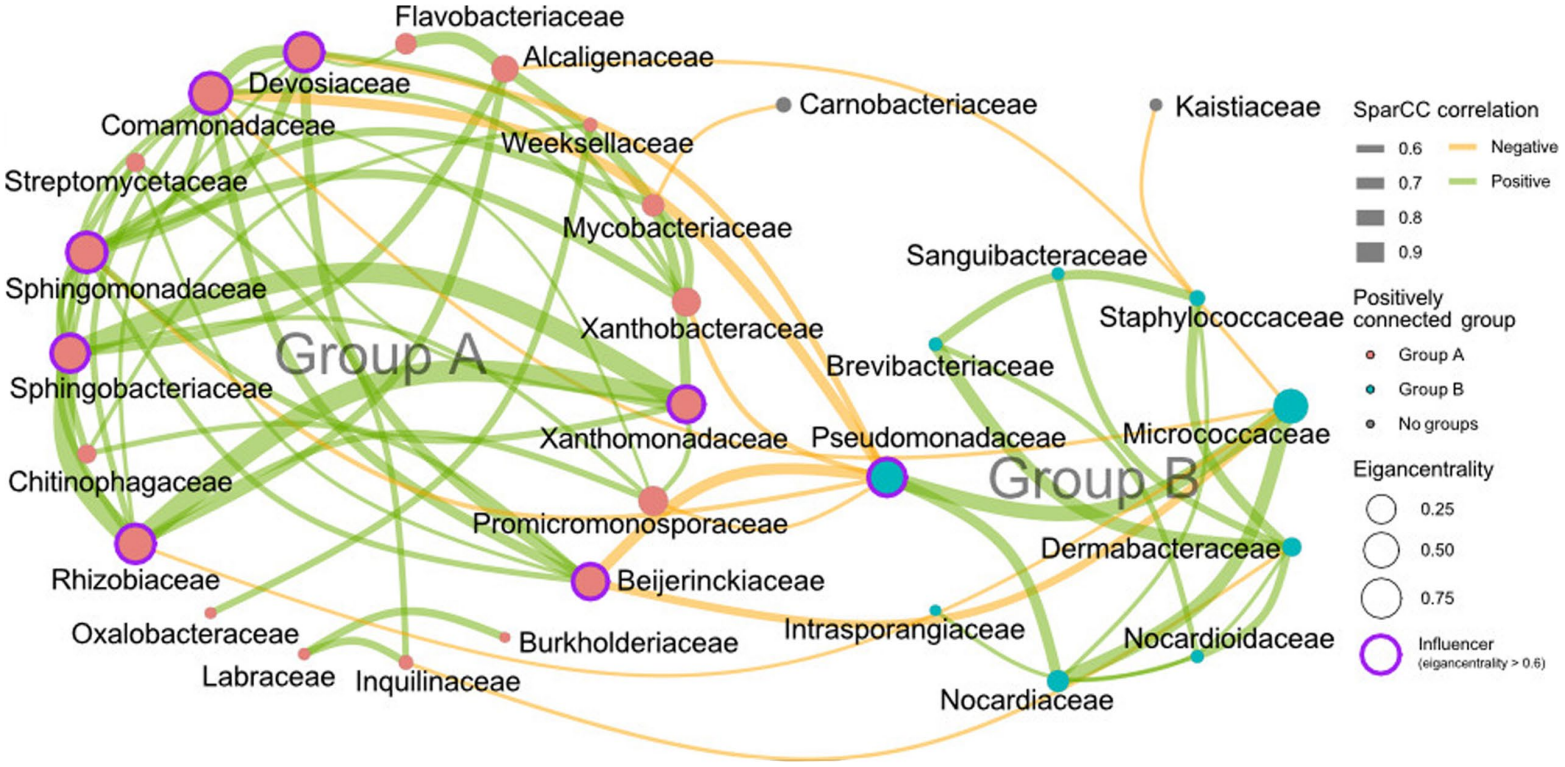
Correlation network analysis in the rhizosphere. The cumulative abundance of the 95% ASV in the rhizosphere, arranged in descending order, is used for analysis. The SparCC network is calculated at the family level. The thickness and color of the edge lines indicate the magnitude of the correlation, with a positive correlation in green and a negative correlation in yellow. The node size represents the eigencentrality, and families with an eigencentrality greater than 0.6 are considered structurally important within the network and thus regarded as influencers. This analysis identifies two distinct groups connected only by positive correlations. The influencers within group A include, Devosiaceae, Comamonadaceae, Sphingomonadaceae, Sphingobacteriaceae, Rhizobiaceae, Beijerinckiaceae, and Xanthomonadaceae, while group B contains only Pseudomonadaceae as its influencer (*p* < 0.01, correlation magnitude >0.5).

**Figure 4:**
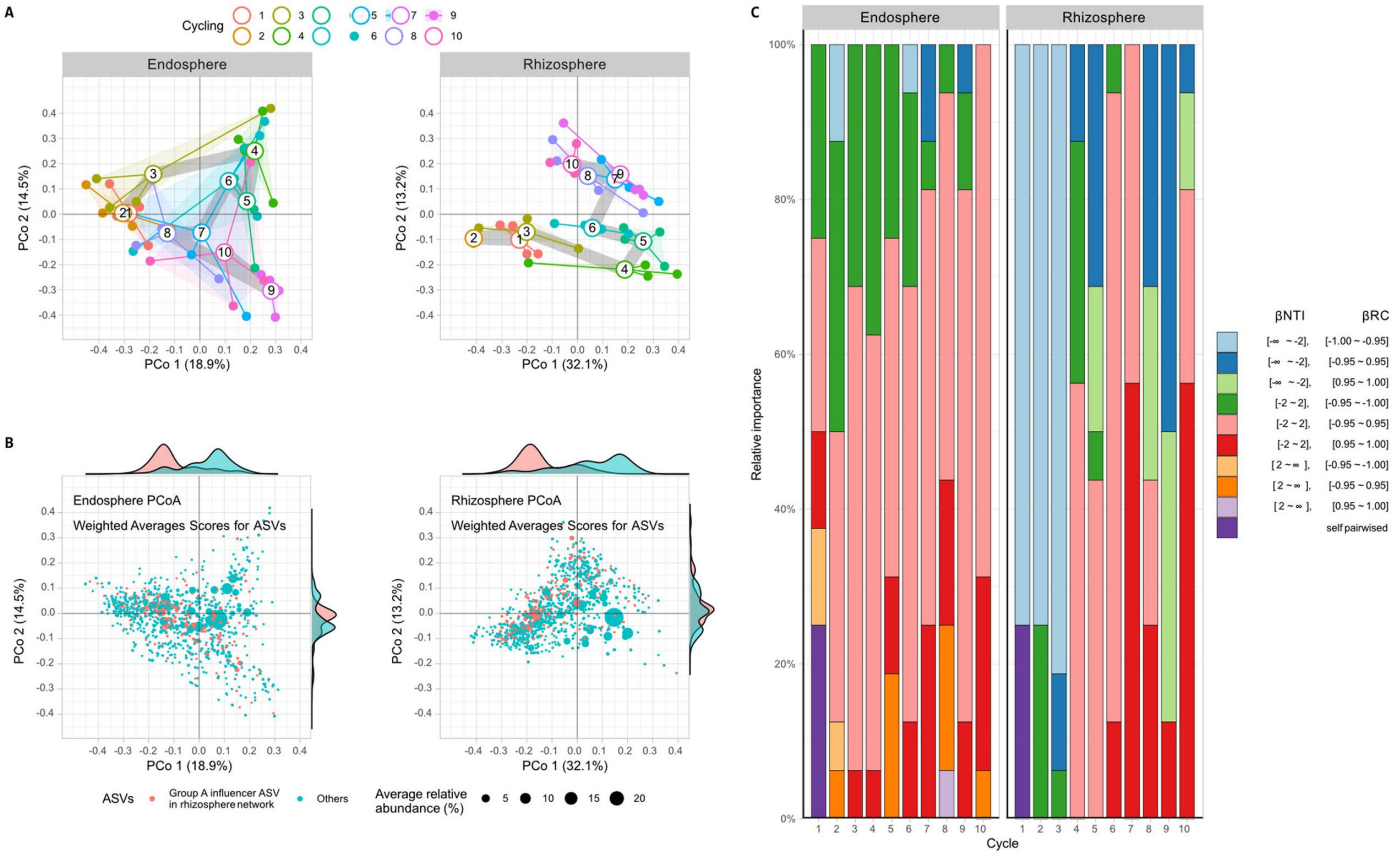
Beta diversity among time points of ginseng monoculture. A principal coordinates analysis (PCoA) is performed using Bray-Curtis distance to analyze the beta diversity in both the endosphere and rhizosphere. **(A)** The PCoA graph shows open circles representing the average of each sample, described by closed circles. The thick grey line represents the path of averages per circle. **(B)** A weighted score analysis is performed about the PCoA results. Each dot represents an ASV, and the position of the points indicates their influence as revealed by the PCoA results. The size of each dot reflects the average relative abundance of the ASVs, with influencers in rhizosphere network group A marked in pink and others in cyan. Additionally, the marginal plot represents the relative abundances weighted distribution of ASVs. **(C)** Stochastic ecological turnover is analyzed by comparing β NTI and β _RC_ values. A β NTI value >2 indicates significant phylogenetic differences, while a value < −2 indicates no significant phylogenetic differences in the cycle 1 bacterial community. A β _RC_ value >0.95 or < −0.95 indicates a significant lack of sharing or sharing of the cycle 1 community composition. These β NTI and β _RC_ values describe the stochastic community turnover phase.

To comprehend the interactions among bacteria and their assembly, a correlation network was calculated using the FastSpar algorithm at the family level and a principal coordinate analysis (PCoA) was conducted. Results revealed a lack of structural regularity in the endosphere network (Supplementary Figure S5), however, two groups of families were connected by positive correlations were discovered in the rhizosphere networks (Figure 3). Group A consisted of 19 families, including Flavobacteriaceae, Alcaligenaceae, Weeksellaceae, Mycobacteriaceae, Xanthobacteraceae, Xanthomonadaceae, Promicromonosporaceae, Beijerinckiaceae, Burkholderiaceae, Inquilinaceae, Labraceae, Oxalobacteraceae, Rhizobiaceae, Chitinophagaceae, Sphingobacteriaceae, Sphingomonadaceae, Streptomycetaceae, Comamonadaceae, and Devosiaceae. Group B consisted of 9 families, including Staphylococcaceae, Micrococcaceae, Dermabacteraceae, Nocardioidaceae, Nocardiaceae, Intrasporangiaceae, Pseudomonadaceae, Brevibacteriaceae, and Sanguibacteriaceae (Figure 3). It also shows that is only a negative connection between group A and B. In the network structure, which includes both positive and negative correlations, Devosiaceae, Comamonadaceae, Sphingomonadaceae, Sphingobacteriaceae, Rhizobiaceae, Beijerinckiaceae, Xanthomonadaceae, and Psudomonadaceae had high influence (eigencentralioty >0.6). The influencers, except Psudomonadaceae, belonged to group A. Given the association between higher rhizosphere alpha diversity and reduced root rot disease (Figures 2D,E), it was hypothesized that group A, which had more members than group B, is important structurally and functionally for the community. To verify this from a community structure perspective, the effect of group A influencers on beta diversity was observed through PCoA analysis (Figure 4A) and analysis of weighted average scores for ASVs (Figure 4B). In the PCoA analysis, both the endosphere and rhizosphere showed fluctuations after cycles 1–3, which was before cycles 4–5, the highest disease index value. Additionally, the analysis of weighted average scores revealed a distinct distribution of group A influencers in the rhizosphere along the PCo1 axis, clearly differentiating them from the other ASVs. These networks and PCoA analyses indicate that influencers in group A within the rhizosphere are crucially important to the community structure.

### Microbiota community turnover and keystone taxa

The beta diversity of bacterial communities in different localities was statistically analyzed (Figure 4). The β NTI and β _RC_ indices were calculated for each pair of samples (Fig S6) and the number of statistically significant pairswas counted to comput and summarize the ratios basis on cycle 1 (Figure 4C). Significantly, the pair of communities is phylogenetically distinguished based on 16S V4 region when β NTI was < −2 and not distinguished when β NTI was >2. A β _RC_ value between −1 and 1 indicated that if it was higher than 0.95, the ASVs of the pair communities were not shared between communities, and if it was lower than −0.95, the ASVs of the pair samples were shared between communities. When considering both β NTI and β _RC_, the changes in the bacterial community were observed in comparison to the first cycle. Based on the first cycle, the state of β NTI < −2 and β _RC_ < −0.95 was primarily observed from the first to the third cycle in the rhizosphere, but this state could not be observed from the fourth to the tenth cycle in the rhizosphere (Figure 4C and Supplementary Figure S6). In the endosphere, the β NTI did not change much compared to the rhizosphere, but the case of β _RC_ < −0.95 decreased after the sixth cycle. The bacterial community turnover was presumed to begin at the fourth cycle, as the changes in the rhizosphere were large and early (Figure 4C and Supplementary Figure S5).

**Figure 5:**
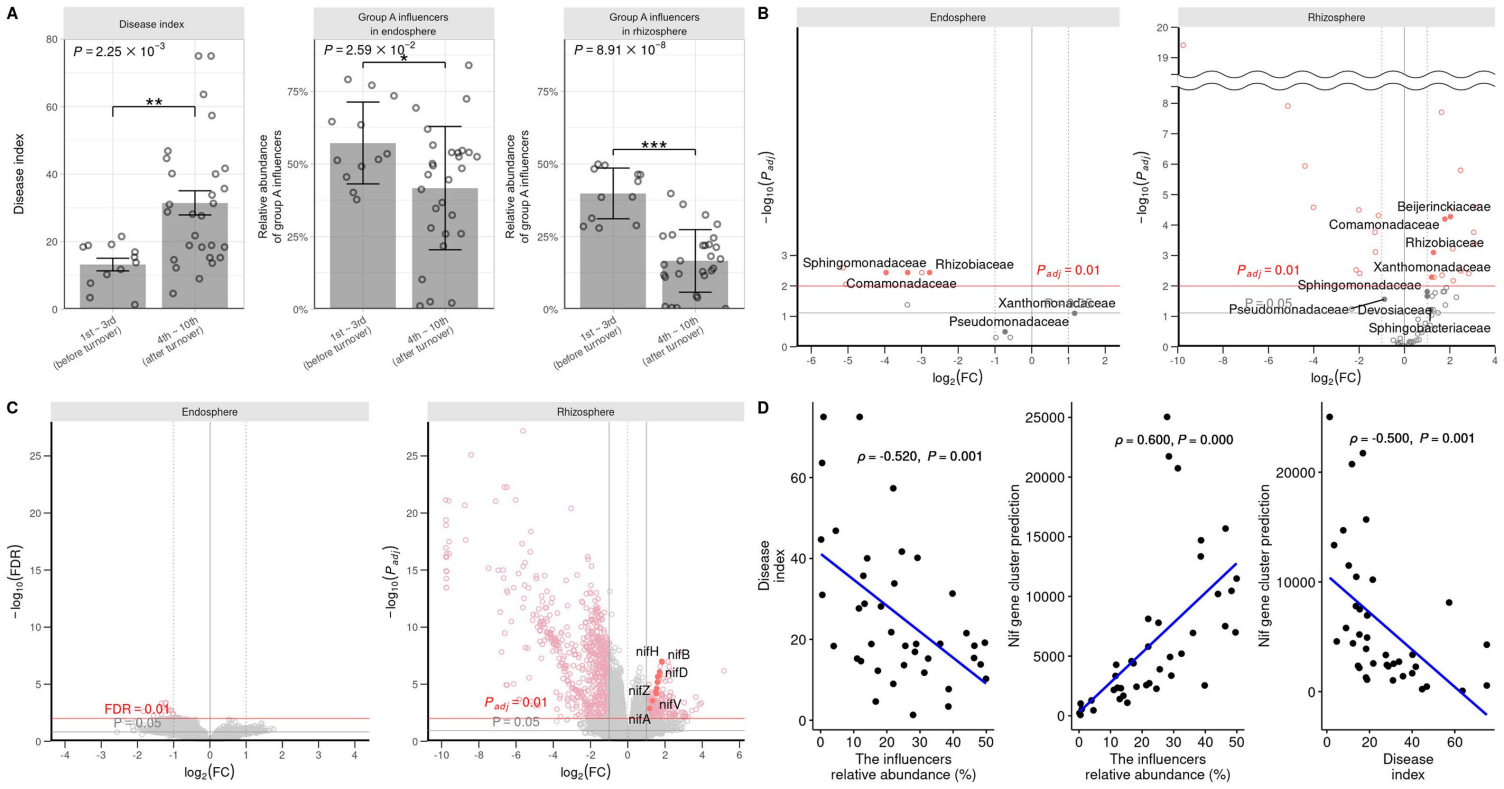
Comparison of root rot disease index, group A influencers, and predicted nitrogen fixation gene expression before and after turnover. **(A)** The disease index and relative abundance of group A influencer taxa in the endosphere and rhizosphere are analyzed by Wilcoxon signed-rank test. Student’s *T*-test (*P ≦ 0.05; **P ≦ 0.01; ***P ≦0.001) is used to analyze the abundance of influencer taxa in the endosphere and rhizosphere. The influencer taxa abundances **(B)** and KEGG ontology **(C)** are predicted by PICRUSt2 and DESeq2. DESeq2 is used to compute volcano plots for the endosphere and rhizosphere. The *P_adj_* values are calculated using the FDR method. Log2fold changes >0 and <0 are shown before and after turnover. **(D)** Pearson correlation analysis is performed to assess the relationship between the influencer taxa abundance of the rhizosphere, the root rot disease index, and the predicted nitrogen fixation. The relationship between the influencer abundance, disease index, and nitrogen fixation-associated gene cluster is analyzed. A positive linear correlation ( ρ approaching 1), no correlation ( ρ approaching 0), and negative linear correlation ( ρ approaching −1) are observed. The abundance and nitrogen fixation ability are negatively correlated with root rot occurrence.

The student’s *t*-test was performed to assess the difference in the progression of root rot and the abundance of group A influential taxa before and after the community turnover (Figure 5A). The results indicated that after the turnover, the progression of root rot was greater, and the abundance of influential taxa was lower in both the rhizosphere and endosphere. Differential analysis of individual ASVs was conducted using DESeq2 and revealed that same group A influencer families Sphingomonadaceae, Comamonadaceae, and Rhizobiaceae, were more prevalent after the turnover in the endosphere. Conversely, some group A influencer Beijgerkiaceae, Commandaceae, Rhizobiaceae, and Xanthomonadaceae were more abundant before the turnover in the rhizosphere (Figure 5B). In summary, these results indicate that influencers in group A play a role in suppressing root rot disease in the rhizosphere community, thus they can be defined as keystone taxa before turnover.

### Nitrogen metabolism of keystone taxa

**Figure 6:**
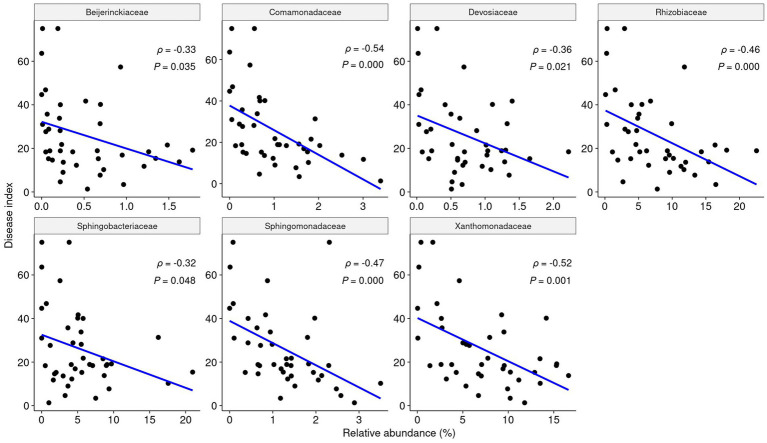
The relative abundance of each influential taxa in the rhizosphere is negatively correlated with root rot disease. According to Pearson analysis, each influencer shows a significant negative linear correlation with the root rot index (*p* < 0.05, ρ < 0).

**Figure 7:**
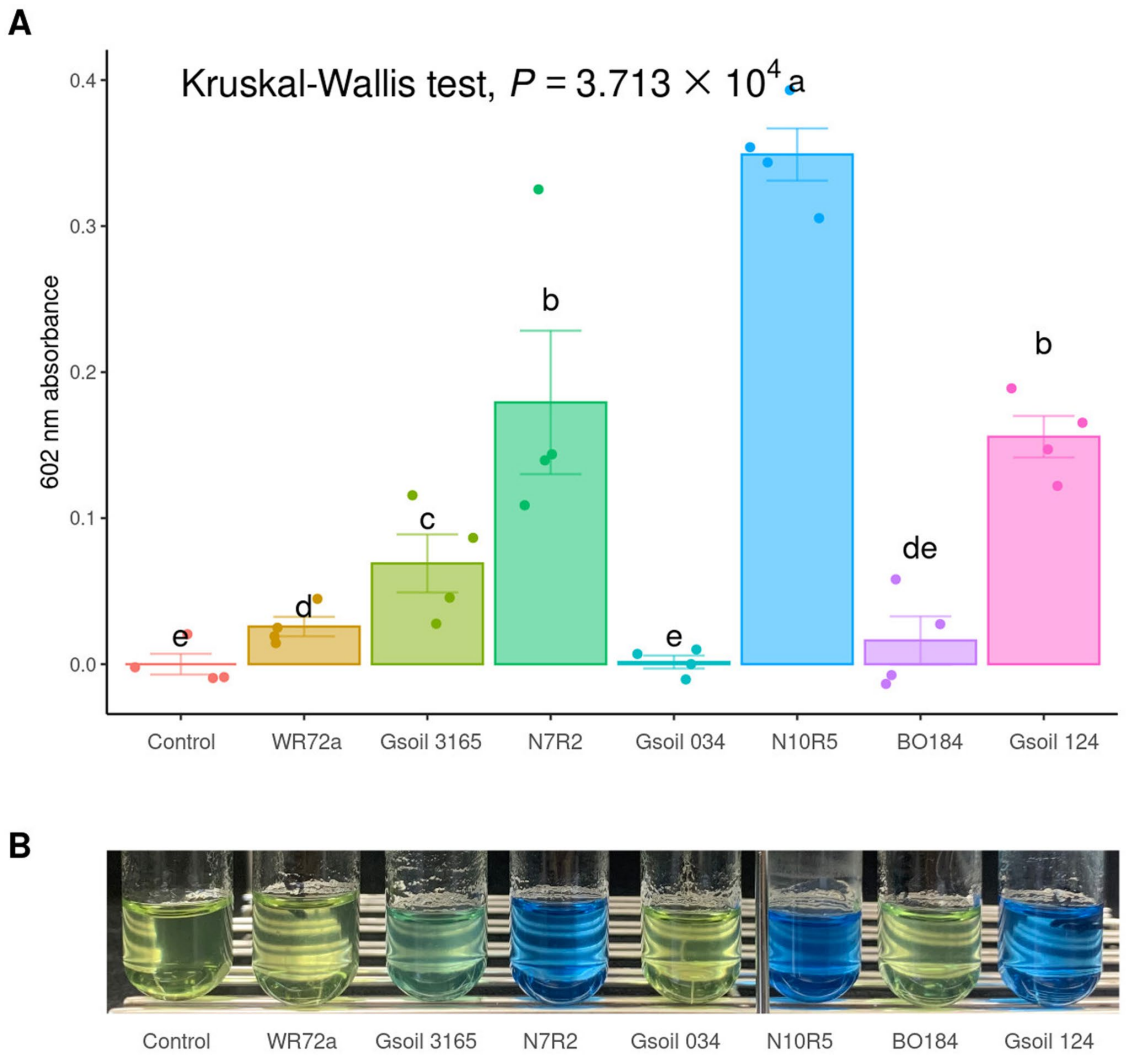
Nitrogen fixation test of isolated and obtained influencers. **(A)** The ability of nitrogen fixation is evaluated by measuring the absorbance at 602 nm after a two-week incubation in semi-solid NFb media. Significantly grouping letters are determined using Conover’s test. **(B)** The color changes in bromothymol blue are observed after 1 month.

To observe how keystone taxa, the group A influencer, suppress disease, the functional differences in the communities before and after turnover were analyzed using the PICRUSt2 workflow. Results showed that in contrast to the endosphere, nitrogen fixation (*nif*) gene clusters were more abundant in the rhizosphere prior to turnover. This was based on the KEGG enzyme commission (EC) number prediction (Figure 5C). A correlation analysis showed that the sum of the predicted *nif* cluster expression tended to increase with the abundance of influential rhizosphere taxa, while it tended to decrease as the root rot disease index increased (Figures 5D, 6). KEGG BRITE functional hierarchies showed that KEGG module ID M00175, describing ‘Nitrogen fixation, nitrogen = > ammonia’ was among the top three before turnover (Supplementary Table S2). To verify the importance of the influential taxa (Table 1) for nitrogen fixation, the nitrogen-fixing ability was measured in Nfb media of the taxa, which were the same as the influential ASV sequences. Results showed that compared to the control (no inoculation), WR72a, Gsoil 3,165, N7R2, N10R5, BO184, and Gsoil 124 had significant nitrogen-fixing ability in semi-solid NFb media (Figure 7). Additionally, PICRUSt2 predictions using the MetaCyc metabolic pathway database showed that most of the amino acid biosynthesis and degradation pathways were more active before turnover than after the event (Supplementary Figures S7, 8).

## Discussion

The occurrence of crop monoculture is known to increase the incidence of specific plant diseases. Despite this, the continuous cultivation of crops using monoculture methods can result in the decline of disease incidents, a phenomenon known as suppression, reported in wheat cropping regions such as North America, Europe, and Australia (Schlatter et al., 2017). This decline in the take-all disease (caused by *Gaemannomyces graminis* var. *tritici*) has been named take-all decline (TAD) and is attributed to the presence of beneficial microbes in the wheat rhizosphere, particularly *Pseudomonas* spp., which inhibit pathogenic fungi through the secretion of antifungal compounds such as phenazine or 2,4-diacetylpholoroglucinol (Kwak and Weller, 2013). However, there has been limited research on the initial microbial populations and soil microbial variability during the period when the incidence of disease explodes in the early stages of monoculture. This study aims to increase our understanding of the variation in the initial microbiota community forming plant-microbe relationships in monoculture crop environments and the increase in disease occurrence. To this end, we have introduced a long-term crop cultivation system using a monoculture model of ginseng and conducted a study of the microbiota before and after the disease outbreak.

The results of qPCR analysis using ITS1 and AFP346 primers and phenotype measurements have revealed that the serial *F. solani* population density changes and its impact on root rot disease occurrences. In general, *F. solani* density was high, reaching up to 10^5^ CFU/g of soil, due to the soil being sourced from a serial ginseng cultivation field. During the ginseng monoculture process in this study, the density decreased from approximately 10^6^ to 10^5^ CFU/g of soil, however, this decrease did not affect the incidence of root rot. Utilizing MiSeq technology to gather information on the microbiota community, it was confirmed that the Shannon index, Simpson index, and Observed ASV representing *α* diversity of the rhizosphere showed a linear correlation with the root rot disease index. On the other hand, the endosphere α diversity was not linearly related to the progression of root rot. These findings suggest that the structure of the rhizosphere microbiota community determines the progression of root rot at a sufficient density of *F. solani*. This is consistent with reports indicating a decrease in rhizosphere bacterial α diversity when plant diseases occurred (Ma et al., 2019). However, it was observed that the endosphere α diversity was more stable than the rhizosphere α diversity during root rot progression, likely due to the direct physiological homeostasis of ginseng.

The SparCC network revealed two distinct groups with negative interactions in the rhizosphere. The groups were determined by cutting off *p*-values under 0.01 and correlative magnitudes above 0.5 at the family level. Group A, comprised of 19 nodes, was more diverse and complex than group B, which consisted of nine nodes. Lower α diversity in the presence of root rot disease indicated that group A constituted a major bacterial community in the rhizosphere due to its positive internal interactions. The influential taxa were selected based on their eigencentrality of 0.6 or higher. The influencers in group A were found to have a negative correlation with the root rot disease index. The network and node properties’ results suggest that these influencer taxa present in the rhizosphere are necessary for preventing or defending against root rot. Moreover, these influencers, except for Pseudomonadaceae, are consistent with the definition of keystone taxa, given their structural importance and crucial roles within the community (Banerjee et al., 2018).

The structure of the microbiota community was statically analyzed to investigate the beta-diversity in-depth, with reference to previous studies (Stegen et al., 2012; Chase et al., 2011; Gao et al., 2020). The analysis was performed before and after the fourth and fifth cycling periods, which were characterized by a severe outbreak of root rot disease. Results showed that prior to the fourth and fifth cycling, the rhizosphere bacterial communities had not experienced an influx of phylogenetically new bacteria ( β NTI < −2) or a change in the distribution of bacteria ( β _RC_ < −0.95) compared to the first cycling period. However, during the fourth and fifth cycling periods or after, the stability of the rhizosphere community sharply decreased. This sudden change was not observed in the endosphere. These findings were consistent with the results of the PCoA, and the weighted score analysis, which showed that abundant ASVs belonging to influential taxa with an abundance greater than 0.1% were intensively distributed in the first to third cycling periods. The distribution of influencers in the rhizosphere revealed that they were more prevalent in the first to third cycling stages as compared to the fourth cycling stage. This was confirmed through a student’s *t*-test and major changes in the influencers were observed in the rhizosphere, including an increase in the abundance of Beijerinckiaceae, Comamonadaceae, Rhizobiaceae, and Xanthomonadaceae. The severe outbreak of root rot was accompanied by a turnover in the microbial community in the rhizosphere, with these changes in the influencer populations being the primary drivers of the turnover. Given that disease-suppressive soil is typically formed following severe outbreaks of the disease (Weller et al., 2002), it can be inferred that the observed turnover, characterized by an influx of microbiota and a reshuffling of the initial bacterial community, is a crucial precursor to the formation of disease-suppressive soil from the perspective of the bacterial community.

The Comamonadaceae, Sphingomonadaceae, and Xanthomonadaceae families have been widely utilized for bioremediation due to their capability for xenobiotic metabolism (Barooah and Sen, 1964). These bacteria are able to help eliminate harmful mycotoxins produced by *Fusarium* (Urbaniak et al., 2020). Taxonomically, the selected influencer taxa belong to the known diazotrophic groups such as Beijernckiacea, Devosiaceae and Rhizobiaceae which are classified under the order Rhizobiales (Barooah and Sen, 1964; Rivas et al., 2002). Xanthomonadaceae, with the genus *Stenotrophomonas* as the most abundant, were also reported to be diazotrophic (Kumar et al., 2019). Both Sphingomonadaceae and Comamonadaceae are capable of nitrogen fixation. Although *Sphingobacter* is not a major nitrogen-fixing bacterium, it has been shown to convert the NH_4_^+^ produced by nitrogen fixation into NO_3_^−^ (Hester et al., 2018). This information suggests that the selected influencer taxa play a crucial role in fixing and utilizing atmospheric nitrogen in the rhizosphere microbial community.

The abundance of rhizosphere influencer taxa was found to be more dominant prior to the turnover. This observation led to the hypothesis that the rhizosphere had a higher capacity for nitrogen fixation and circulation before the turnover. To test this hypothesis, the total number of genes predicted with PICRUSt2 was analyzed. Out of the isolated or distributed bacteria that 100% matched the 16S rRNA V4 region ASV belonging to the influencer taxa, six out of seven were found to have the ability to fix nitrogen. The presence of nitrogen fixation gene clusters was also more prevalent in the rhizosphere prior to the turnover. Furthermore, the relative abundance of the influencers was positively correlated with nitrogen fixation. Additionally, the abundance of influential taxa and *nif* genes showed a negative correlation with the root rot disease index. To further understand the role of nitrogen in the rhizosphere community, PICRUSt2 was utilized to analyze pathways for the biosynthesis or degradation of amino acids, which are commonly metabolized from nitrogen in living organisms. The results suggest that most of the biosynthesis or degradation pathways were more active prior to the turnover. These findings suggest that the rhizosphere influencer taxa may have had an unknown disease suppression mechanism triggered by nitrogen fixation. Further research is needed to determine the specific nitrogen metabolism of the influencer and correlative taxa and how it affects the characteristics of the rhizosphere community, as previous reports have shown that bacterial communities can be impacted by nitrogen fertilization (Lv et al., 2017). This initial community, similar to those of general suppressive soils, is controlled by the influencer and suppresses disease through direct or indirect mechanisms triggered by a variety of microbes (Schlatter et al., 2017).

**Figure 8:**
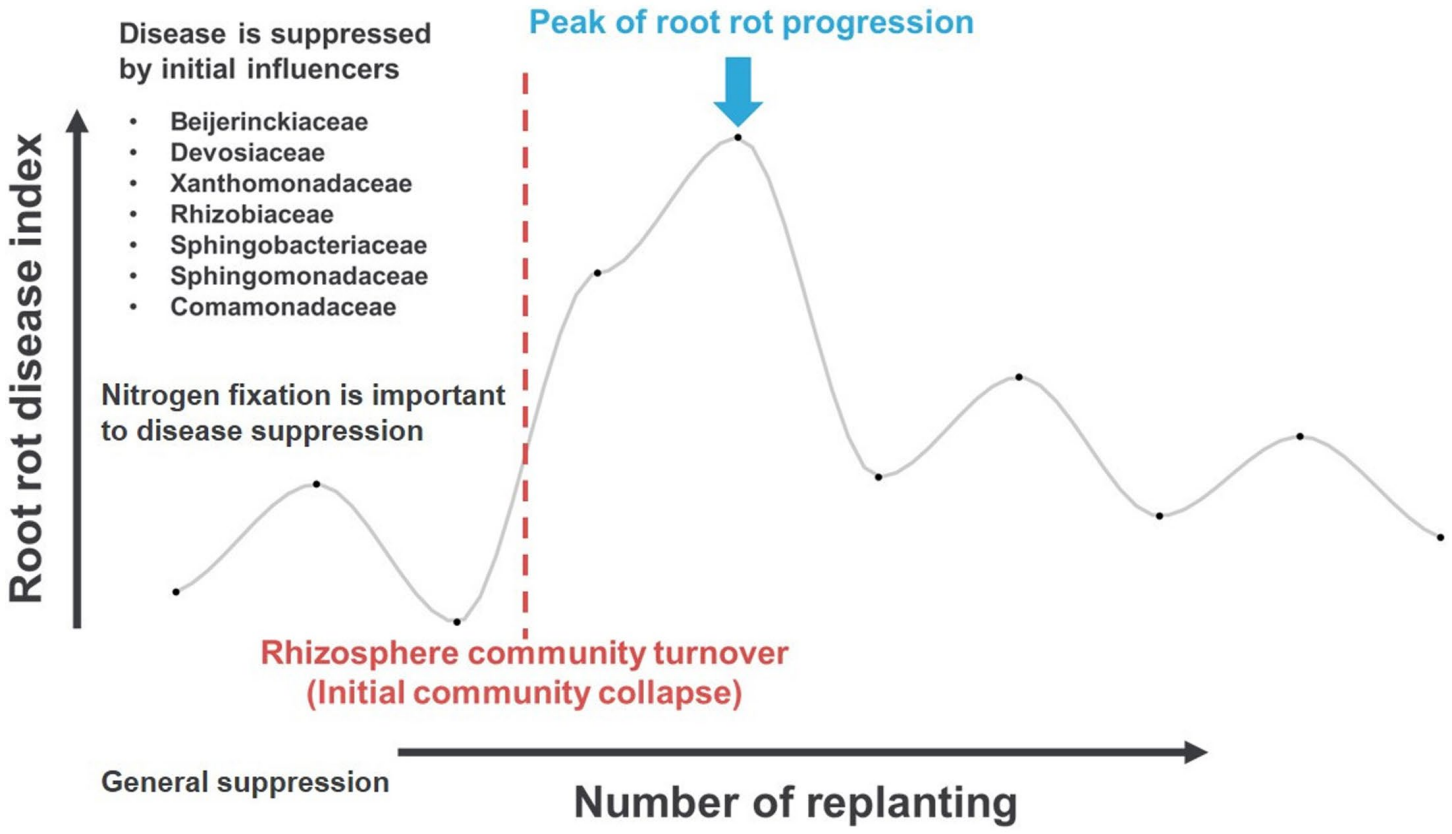
The proposed hypothesis of the initial stage of ginseng monoculture, conducive root rot disease, microbiota shifting, and influencer taxa before turnover.

The impact of shifting microbiota community structure and keystone taxa on plant health at the early stage of monoculture systems has yet to be fully explored. In order to better understand this relationship, this study was designed to monitor the shifting microbiota community structure using a continuous monoculture model system of ginseng as a model plant. The findings showed that the changes in the microbiota community were more pronounced in the rhizosphere than in the endosphere, likely due to the use of ginseng from the seedling stage with a consistent aging factor. In the rhizosphere, an early community collapse was observed between the third and fourth plantings, prior to the fifth planting when root rot progressed the most. The initial community was believed to be regulated by certain influencers, which facilitate nitrogen circulation through nitrogen fixation. The early-stage communities, regulated by these influencers, suppressed disease in ways that are not yet understood through nitrogen circulation (Figure 8).

## Data Availability

The datasets presented in this study can be found in online repositories. The names of the repository/repositories and accession number(s) can be found at: https://www.ncbi.nlm.nih.gov/, PRJNA971581.

## Footnotes

^1^ https://github.com/vegandevs/vegan

^2^ https://github.com/joey711/phyloseq

^3^ https://github.com/scwatts/fastspar

^4^ https://github.com/mikelove/DESeq2

^5^ https://github.com/tidyverse/ggplot2

^6^ https://github.com/gyeongjunCho/R-code-of-Ph.D.-Thesis
