# Supplementary material for: Ecological shifts in soil microbiota and root rot disease progress during ginseng monoculture
Source: Front Microbiol. 2024 Oct 18;15:1442208. doi: 10.3389/fmicb.2024.1442208 (PMC11530993; doi:10.3389/fmicb.2024.1442208)
Supplement: Supplementary file 1 [file Data_Sheet_1.docx]

**Supplementary Information**

*for*

**Ecological Shifts in Soil Microbiota and Root rot Disease Progress during Ginseng Monoculture**

Gyeongjun Cho, Da-Ran Kim, and Youn-Sig Kwak

This file includes 2 tables and 8 figures

**Table S1.** Reads quality control results

| **Sample** | **Organization** | **Raw forward** | **Raw reverse** | **Filtered forward** | **Filtered reverse** | **Denoised forward** | **Denoised reverse** | **Merged reads** | **Removed chimera** | **Only bacteria** |
| --- | --- | --- | --- | --- | --- | --- | --- | --- | --- | --- |
| EnC1P1 | Endosphere | 94,223 | 94,223 | 70,691 | 70,691 | 69,118 | 69,640 | 62,240 | 38,669 | 37,227 |
| EnC1P3 | Endosphere | 97,030 | 97,030 | 76,120 | 76,120 | 75,313 | 75,862 | 73,083 | 49,124 | 41,378 |
| EnC1P5 | Endosphere | 93,936 | 93,936 | 82,779 | 82,779 | 81,588 | 82,064 | 73,092 | 54,947 | 53,909 |
| EnC1P7 | Endosphere | 83,466 | 83,466 | 79,653 | 79,653 | 78,591 | 79,117 | 74,382 | 50,827 | 46,616 |
| EnC2P1 | Endosphere | 82,781 | 82,781 | 59,742 | 59,742 | 58,732 | 59,175 | 54,771 | 37,653 | 37,007 |
| EnC2P3 | Endosphere | 90,562 | 90,562 | 81,687 | 81,687 | 80,711 | 81,087 | 75,494 | 54,638 | 51,477 |
| EnC2P5 | Endosphere | 100,295 | 100,295 | 73,580 | 73,580 | 72,928 | 73,364 | 69,831 | 48,686 | 43,550 |
| EnC2P7 | Endosphere | 94,031 | 94,031 | 79,639 | 79,639 | 78,687 | 79,179 | 74,249 | 48,064 | 45,824 |
| EnC3P1 | Endosphere | 70,705 | 70,705 | 65,650 | 65,650 | 65,172 | 65,473 | 62,368 | 40,770 | 30,426 |
| EnC3P3 | Endosphere | 96,983 | 96,983 | 82,870 | 82,870 | 82,373 | 82,540 | 79,905 | 58,710 | 44,357 |
| EnC3P5 | Endosphere | 87,382 | 87,382 | 69,486 | 69,486 | 68,603 | 69,035 | 64,793 | 42,308 | 40,187 |
| EnC3P7 | Endosphere | 94,484 | 94,484 | 76,274 | 76,274 | 75,185 | 75,574 | 70,036 | 44,755 | 33,763 |
| EnC4P1 | Endosphere | 78,452 | 78,452 | 76,689 | 76,689 | 75,694 | 76,119 | 69,037 | 41,346 | 41,224 |
| EnC4P3 | Endosphere | 96,678 | 96,678 | 72,703 | 72,703 | 72,304 | 72,583 | 70,651 | 51,758 | 31,798 |
| EnC4P5 | Endosphere | 82,062 | 82,062 | 73,892 | 73,892 | 73,426 | 73,706 | 71,438 | 50,783 | 34,997 |
| EnC4P7 | Endosphere | 88,884 | 88,884 | 54,855 | 54,855 | 54,277 | 54,633 | 51,857 | 32,377 | 20,920 |
| EnC5P1 | Endosphere | 90,020 | 90,020 | 83,152 | 83,152 | 82,083 | 82,533 | 77,611 | 51,999 | 28,596 |
| EnC5P3 | Endosphere | 87,744 | 87,744 | 79,709 | 79,709 | 78,427 | 79,073 | 72,936 | 50,095 | 46,416 |
| EnC5P5 | Endosphere | 87,334 | 87,334 | 72,449 | 72,449 | 71,984 | 72,143 | 69,419 | 52,866 | 30,942 |
| EnC5P7 | Endosphere | 69,553 | 69,553 | 77,791 | 77,791 | 77,113 | 77,268 | 72,600 | 48,044 | 29,687 |
| EnC6P1 | Endosphere | 99,764 | 99,764 | 76,011 | 76,011 | 75,093 | 75,445 | 68,977 | 45,966 | 45,615 |
| EnC6P3 | Endosphere | 94,196 | 94,196 | 77,086 | 77,086 | 76,012 | 76,301 | 71,729 | 43,577 | 37,111 |
| EnC6P5 | Endosphere | 90,777 | 90,777 | 77,975 | 77,975 | 77,298 | 77,661 | 73,752 | 51,969 | 39,210 |
| EnC6P7 | Endosphere | 92,472 | 92,472 | 81,481 | 81,481 | 80,536 | 81,066 | 76,360 | 50,348 | 31,430 |
| EnC7P1 | Endosphere | 89,109 | 89,109 | 59,437 | 59,437 | 58,740 | 58,932 | 54,769 | 40,421 | 39,721 |
| EnC7P3 | Endosphere | 91,180 | 91,180 | 67,607 | 67,607 | 67,104 | 67,338 | 64,914 | 43,063 | 25,633 |
| EnC7P5 | Endosphere | 93,285 | 93,285 | 86,813 | 86,813 | 85,175 | 85,857 | 76,836 | 49,673 | 49,089 |
| EnC7P7 | Endosphere | 97,127 | 97,127 | 75,505 | 75,505 | 74,315 | 74,844 | 69,218 | 48,206 | 46,102 |
| EnC8P1 | Endosphere | 70,558 | 70,558 | 74,353 | 74,353 | 73,435 | 73,909 | 69,106 | 45,281 | 43,857 |
| EnC8P3 | Endosphere | 79,636 | 79,636 | 62,483 | 62,483 | 61,249 | 61,737 | 56,901 | 38,499 | 36,913 |
| EnC8P5 | Endosphere | 101,809 | 101,809 | 81,432 | 81,432 | 79,223 | 80,047 | 71,030 | 47,330 | 41,210 |
| EnC8P7 | Endosphere | 88,632 | 88,632 | 67,284 | 67,284 | 65,856 | 66,353 | 60,128 | 38,073 | 37,236 |
| EnC9P1 | Endosphere | 87,970 | 87,970 | 71,308 | 71,308 | 70,412 | 70,887 | 66,176 | 47,191 | 43,962 |
| EnC9P3 | Endosphere | 73,766 | 73,766 | 68,059 | 68,059 | 67,496 | 67,696 | 64,229 | 41,463 | 34,858 |
| EnC9P5 | Endosphere | 96,767 | 96,767 | 83,764 | 83,764 | 82,555 | 83,020 | 77,495 | 54,069 | 51,612 |
| EnC9P7 | Endosphere | 80,064 | 80,064 | 63,746 | 63,746 | 63,121 | 63,391 | 59,690 | 36,073 | 31,692 |
| EnC10P1 | Endosphere | 84,923 | 84,923 | 79,533 | 79,533 | 78,615 | 78,950 | 74,982 | 56,790 | 56,747 |
| EnC10P3 | Endosphere | 79,451 | 79,451 | 83,666 | 83,666 | 82,582 | 83,045 | 78,223 | 55,374 | 55,136 |
| EnC10P5 | Endosphere | 98,079 | 98,079 | 77,236 | 77,236 | 76,540 | 76,846 | 71,517 | 43,523 | 41,888 |
| EnC10P7 | Endosphere | 76,183 | 76,183 | 69,045 | 69,045 | 68,283 | 68,478 | 64,696 | 45,257 | 39,951 |
| RhC1P1 | Rhizosphere | 125,360 | 125,360 | 124,674 | 124,674 | 122,422 | 123,038 | 115,449 | 98,596 | 93,479 |
| RhC1P3 | Rhizosphere | 122,819 | 122,819 | 101,488 | 101,488 | 98,538 | 99,428 | 91,561 | 80,147 | 71,096 |
| RhC1P5 | Rhizosphere | 129,538 | 129,538 | 100,775 | 100,775 | 97,725 | 98,338 | 90,007 | 78,095 | 68,978 |
| RhC1P7 | Rhizosphere | 124,641 | 124,641 | 91,363 | 91,363 | 89,765 | 90,131 | 84,469 | 72,060 | 60,898 |
| RhC2P1 | Rhizosphere | 156,541 | 156,541 | 101,767 | 101,767 | 99,781 | 100,154 | 93,076 | 78,674 | 75,502 |
| RhC2P3 | Rhizosphere | 128,563 | 128,563 | 71,982 | 71,982 | 70,320 | 70,498 | 60,512 | 53,374 | 47,567 |
| RhC2P5 | Rhizosphere | 128,269 | 128,269 | 92,743 | 92,743 | 90,222 | 91,125 | 80,776 | 70,116 | 62,165 |
| RhC2P7 | Rhizosphere | 116,033 | 116,033 | 102,938 | 102,938 | 100,499 | 101,147 | 89,916 | 76,912 | 68,538 |
| RhC3P1 | Rhizosphere | 130,477 | 130,477 | 94,824 | 94,824 | 93,038 | 93,444 | 87,558 | 75,795 | 66,102 |
| RhC3P3 | Rhizosphere | 104,770 | 104,770 | 110,362 | 110,362 | 108,343 | 108,812 | 100,533 | 85,556 | 79,391 |
| RhC3P5 | Rhizosphere | 114,986 | 114,986 | 92,438 | 92,438 | 90,353 | 90,795 | 83,990 | 69,807 | 64,945 |
| RhC3P7 | Rhizosphere | 129,378 | 129,378 | 102,604 | 102,604 | 100,612 | 101,315 | 95,274 | 77,940 | 70,445 |
| RhC4P1 | Rhizosphere | 122,435 | 122,435 | 107,254 | 107,254 | 105,827 | 106,404 | 100,893 | 82,628 | 61,679 |
| RhC4P3 | Rhizosphere | 139,571 | 139,571 | 88,242 | 88,242 | 87,706 | 87,957 | 85,380 | 65,678 | 33,796 |
| RhC4P5 | Rhizosphere | 116,129 | 116,129 | 99,665 | 99,665 | 98,986 | 99,119 | 96,283 | 75,039 | 47,398 |
| RhC4P7 | Rhizosphere | 129,665 | 129,665 | 87,436 | 87,436 | 86,818 | 86,951 | 83,821 | 63,154 | 39,386 |
| RhC5P1 | Rhizosphere | 136,800 | 136,800 | 100,825 | 100,825 | 99,455 | 99,931 | 94,198 | 77,962 | 51,403 |
| RhC5P3 | Rhizosphere | 111,586 | 111,586 | 91,920 | 91,920 | 91,008 | 91,355 | 87,380 | 69,121 | 49,457 |
| RhC5P5 | Rhizosphere | 123,821 | 123,821 | 90,545 | 90,545 | 89,612 | 89,895 | 85,441 | 68,396 | 47,851 |
| RhC5P7 | Rhizosphere | 110,120 | 110,120 | 105,454 | 105,454 | 103,809 | 104,283 | 96,925 | 77,777 | 57,237 |
| RhC6P1 | Rhizosphere | 130,438 | 130,438 | 111,407 | 111,407 | 109,132 | 109,744 | 101,677 | 83,340 | 77,853 |
| RhC6P3 | Rhizosphere | 117,561 | 117,561 | 107,062 | 107,062 | 104,883 | 105,579 | 97,814 | 77,191 | 73,226 |
| RhC6P5 | Rhizosphere | 114,100 | 114,100 | 89,917 | 89,917 | 87,909 | 88,531 | 82,385 | 66,234 | 60,633 |
| RhC6P7 | Rhizosphere | 131,929 | 131,929 | 91,045 | 91,045 | 89,741 | 89,937 | 84,934 | 69,136 | 43,616 |
| RhC7P1 | Rhizosphere | 144,510 | 144,510 | 106,607 | 106,607 | 105,074 | 105,360 | 100,082 | 83,863 | 81,331 |
| RhC7P3 | Rhizosphere | 136,064 | 136,064 | 106,261 | 106,261 | 104,384 | 104,800 | 98,407 | 81,590 | 80,331 |
| RhC7P5 | Rhizosphere | 113,040 | 113,040 | 97,436 | 97,436 | 95,444 | 95,899 | 89,864 | 75,630 | 74,265 |
| RhC7P7 | Rhizosphere | 112,755 | 112,755 | 94,512 | 94,512 | 92,878 | 93,309 | 88,146 | 74,098 | 73,094 |
| RhC8P1 | Rhizosphere | 133,738 | 133,738 | 125,630 | 125,630 | 123,476 | 123,624 | 116,562 | 97,618 | 95,986 |
| RhC8P3 | Rhizosphere | 136,161 | 136,161 | 99,437 | 99,437 | 97,231 | 97,731 | 90,641 | 75,050 | 73,206 |
| RhC8P5 | Rhizosphere | 120,503 | 120,503 | 118,515 | 118,515 | 116,586 | 117,098 | 110,609 | 92,414 | 91,059 |
| RhC8P7 | Rhizosphere | 117,255 | 117,255 | 101,302 | 101,302 | 99,832 | 100,037 | 95,025 | 80,288 | 76,828 |
| RhC9P1 | Rhizosphere | 159,665 | 159,665 | 91,919 | 91,919 | 90,493 | 90,853 | 86,232 | 73,494 | 68,935 |
| RhC9P3 | Rhizosphere | 126,545 | 126,545 | 95,755 | 95,755 | 94,243 | 94,553 | 89,625 | 77,333 | 72,463 |
| RhC9P5 | Rhizosphere | 148,187 | 148,187 | 113,361 | 113,361 | 110,802 | 111,502 | 103,793 | 86,259 | 79,653 |
| RhC9P7 | Rhizosphere | 125,017 | 125,017 | 100,713 | 100,713 | 99,287 | 99,793 | 95,364 | 79,902 | 76,902 |
| RhC10P1 | Rhizosphere | 118,774 | 118,774 | 97,792 | 97,792 | 95,711 | 96,173 | 89,543 | 74,609 | 73,439 |
| RhC10P3 | Rhizosphere | 121,843 | 121,843 | 97,224 | 97,224 | 94,870 | 95,382 | 88,021 | 74,059 | 71,358 |
| RhC10P5 | Rhizosphere | 145,070 | 145,070 | 102,123 | 102,123 | 99,817 | 100,406 | 92,737 | 75,384 | 63,708 |
| RhC10P7 | Rhizosphere | 126,052 | 126,052 | 99,580 | 99,580 | 97,588 | 97,903 | 90,697 | 74,493 | 72,704 |

**Table S2.** KEGG BRITE functional hierarchy analysis about PICRUSt2 KEGG ontology prediction of turnover before and after in rhizosphere after DESeq2 results (FDR *P_adj_*, < 0.05)

| **Module ID** | **Module description** | **before hit** | **after hit** |
| --- | --- | --- | --- |
| M00539 | Cumate degradation, p-cumate => 2-oxopent-4-enoate + 2-methylpropanoate | 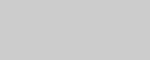 4 | 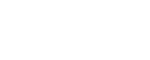 0 |
| M00777 | Avermectin biosynthesis, 2-methylbutanoyl-CoA/isobutyryl-CoA => 6,8a-Seco-6,8a-deoxy-5-oxoavermectin 1a/1b aglycone => avermectin A1a/B1a/A1b/B1b | 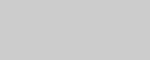 4 | 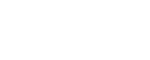 0 |
| M00175 | Nitrogen fixation, nitrogen => ammonia | 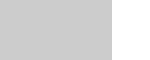 3 | 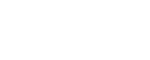 0 |
| M00595 | Thiosulfate oxidation by SOX complex, thiosulfate => sulfate | 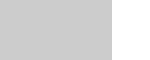 3 | 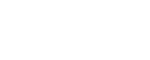 0 |
| M00664 | Nodulation | 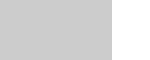 3 | 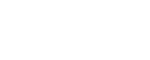 0 |
| M00740 | Methylaspartate cycle | 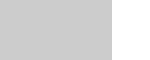 3 | 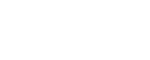 0 |
| M00819 | Pentalenolactone biosynthesis, farnesyl-PP => pentalenolactone | 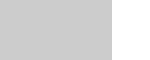 3 | 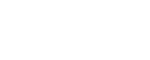 0 |
| M00078 | Heparan sulfate degradation | 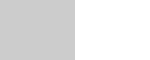 2 | 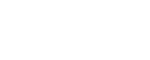 0 |
| M00531 | Assimilatory nitrate reduction, nitrate => ammonia | 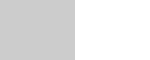 2 | 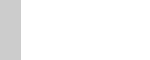 1 |
| M00597 | Anoxygenic photosystem II | 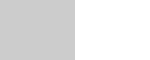 2 | 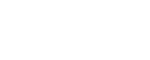 0 |
| M00615 | Nitrate assimilation | 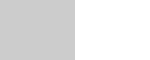 2 | 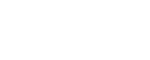 0 |
| M00698 | Multidrug resistance, efflux pump BpeEF-OprC | 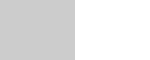 2 | 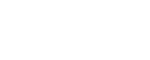 0 |
| M00846 | Siroheme biosynthesis, glutamate => siroheme | 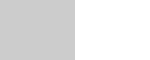 2 | 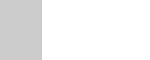 2 |
| M00009 | Citrate cycle (TCA cycle, Krebs cycle) | 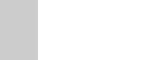 1 | 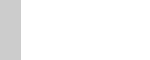 1 |
| M00011 | Citrate cycle, second carbon oxidation, 2-oxoglutarate => oxaloacetate | 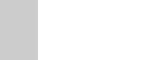 1 | 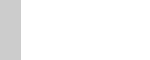 1 |
| M00030 | Lysine biosynthesis, AAA pathway, 2-oxoglutarate => 2-aminoadipate => lysine | 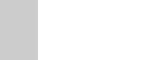 1 | 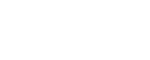 0 |
| M00045 | Histidine degradation, histidine => N-formiminoglutamate => glutamate | 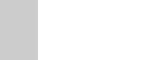 1 | 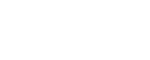 0 |
| M00076 | Dermatan sulfate degradation | 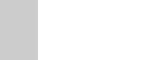 1 | 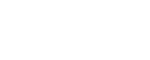 0 |
| M00079 | Keratan sulfate degradation | 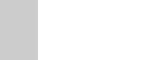 1 | 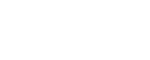 0 |
| M00088 | Ketone body biosynthesis, acetyl-CoA => acetoacetate/3-hydroxybutyrate/acetone | 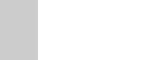 1 | 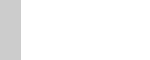 1 |
| M00091 | Phosphatidylcholine (PC) biosynthesis, PE => PC | 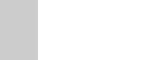 1 | 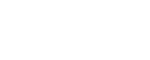 0 |
| M00112 | Tocopherol/tocotorienol biosynthesis | 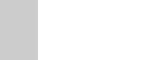 1 | 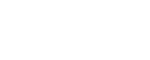 0 |
| M00121 | Heme biosynthesis, plants and bacteria, glutamate => heme | 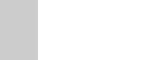 1 | 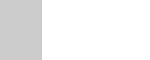 2 |
| M00151 | Cytochrome bc1 complex respiratory unit | 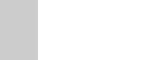 1 | 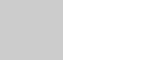 3 |
| M00155 | Cytochrome c oxidase, prokaryotes | 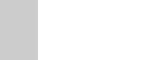 1 | 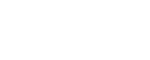 0 |
| M00173 | Reductive citrate cycle (Arnon-Buchanan cycle) | 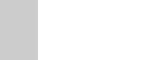 1 | 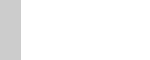 1 |
| M00174 | Methane oxidation, methanotroph, methane => formaldehyde | 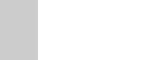 1 | 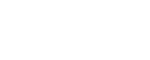 0 |
| M00540 | Benzoate degradation, cyclohexanecarboxylic acid =>pimeloyl-CoA | 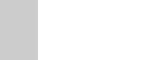 1 | 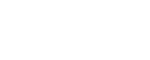 0 |
| M00546 | Purine degradation, xanthine => urea | 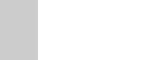 1 | 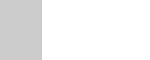 2 |
| M00620 | Incomplete reductive citrate cycle, acetyl-CoA => oxoglutarate | 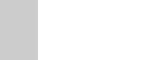 1 | 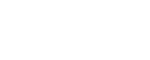 0 |
| M00778 | Type II polyketide backbone biosynthesis, acyl-CoA + malonyl-CoA => polyketide | 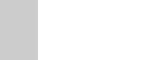 1 | 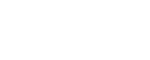 0 |
| M00779 | Dihydrokalafungin biosynthesis, octaketide => dihydrokalafungin | 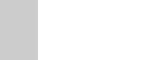 1 | 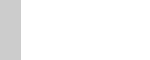 1 |
| M00785 | Cycloserine biosynthesis, arginine/serine => cycloserine | 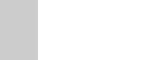 1 | 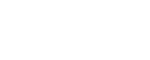 0 |
| M00835 | Pyocyanine biosynthesis, chorismate => pyocyanine | 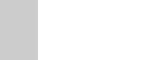 1 | 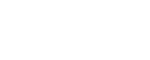 0 |
| M00877 | Kanosamine biosynthesis, glucose 6-phosphate => kanosamine | 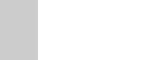 1 | 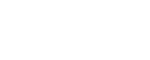 0 |
| M00001 | Glycolysis (Embden-Meyerhof pathway), glucose => pyruvate | 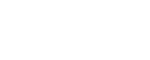 0 | 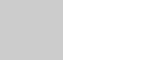 3 |
| M00003 | Gluconeogenesis, oxaloacetate => fructose-6P | 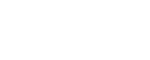 0 | 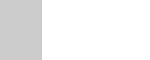 2 |
| M00013 | Malonate semialdehyde pathway, propanoyl-CoA => acetyl-CoA | 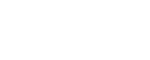 0 | 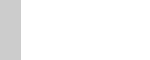 1 |
| M00014 | Glucuronate pathway (uronate pathway) | 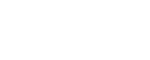 0 | 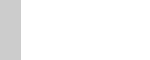 1 |
| M00017 | Methionine biosynthesis, apartate => homoserine => methionine | 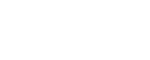 0 | 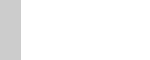 1 |
| M00022 | Shikimate pathway, phosphoenolpyruvate + erythrose-4P => chorismate | 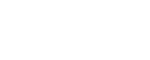 0 | 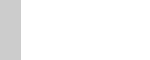 1 |
| M00023 | Tryptophan biosynthesis, chorismate => tryptophan | 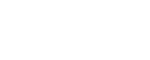 0 | 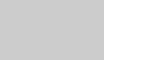 5 |
| M00024 | Phenylalanine biosynthesis, chorismate => phenylpyruvate => phenylalanine | 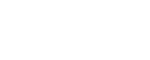 0 | 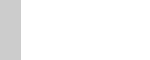 1 |
| M00025 | Tyrosine biosynthesis, chorismate => HPP => tyrosine | 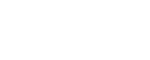 0 | 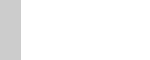 1 |
| M00026 | Histidine biosynthesis, PRPP => histidine | 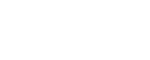 0 | 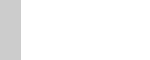 1 |
| M00027 | GABA (gamma-Aminobutyrate) shunt | 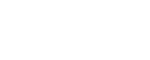 0 | 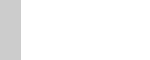 1 |
| M00028 | Ornithine biosynthesis, glutamate => ornithine | 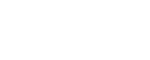 0 | 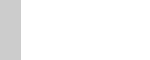 1 |
| M00051 | Uridine monophosphate biosynthesis, glutamine (+ PRPP) => UMP | 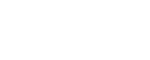 0 | 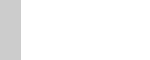 1 |
| M00061 | D-Glucuronate degradation, D-glucuronate => pyruvate + D-glyceraldehyde 3P | 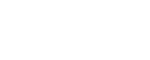 0 | 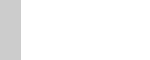 1 |
| M00082 | Fatty acid biosynthesis, initiation | 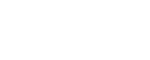 0 | 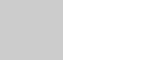 3 |
| M00083 | Fatty acid biosynthesis, elongation | 0 | 1 |
| M00087 | beta-Oxidation | 0 | 1 |
| M00090 | Phosphatidylcholine (PC) biosynthesis, choline => PC | 0 | 1 |
| M00094 | Ceramide biosynthesis | 0 | 1 |
| M00095 | C5 isoprenoid biosynthesis, mevalonate pathway | 0 | 5 |
| M00099 | Sphingosine biosynthesis | 0 | 2 |
| M00101 | Cholesterol biosynthesis, squalene 2,3-epoxide => cholesterol | 0 | 1 |
| M00102 | Ergocalciferol biosynthesis, squalene 2,3-epoxide => ergosterol/ergocalciferol | 0 | 1 |
| M00113 | Jasmonic acid biosynthesis | 0 | 1 |
| M00115 | NAD biosynthesis, aspartate => quinolinate => NAD | 0 | 1 |
| M00116 | Menaquinone biosynthesis, chorismate => menaquinol | 0 | 7 |
| M00120 | Coenzyme A biosynthesis, pantothenate => CoA | 0 | 1 |
| M00126 | Tetrahydrofolate biosynthesis, GTP => THF | 0 | 1 |
| M00127 | Thiamine biosynthesis, prokaryotes, AIR (+ DXP/tyrosine) => TMP/TPP | 0 | 1 |
| M00129 | Ascorbate biosynthesis, animals, glucose-1P => ascorbate | 0 | 1 |
| M00140 | C1-unit interconversion, prokaryotes | 0 | 1 |
| M00150 | Fumarate reductase, prokaryotes | 0 | 1 |
| M00165 | Reductive pentose phosphate cycle (Calvin cycle) | 0 | 1 |
| M00167 | Reductive pentose phosphate cycle, glyceraldehyde-3P => ribulose-5P | 0 | 1 |
| M00345 | Formaldehyde assimilation, ribulose monophosphate pathway | 0 | 2 |
| M00346 | Formaldehyde assimilation, serine pathway | 0 | 1 |
| M00358 | Coenzyme M biosynthesis | 0 | 2 |
| M00364 | C10-C20 isoprenoid biosynthesis, bacteria | 0 | 1 |
| M00365 | C10-C20 isoprenoid biosynthesis, archaea | 0 | 1 |
| M00377 | Reductive acetyl-CoA pathway (Wood-Ljungdahl pathway) | 0 | 1 |
| M00378 | F420 biosynthesis | 0 | 1 |
| M00416 | Cytochrome aa3-600 menaquinol oxidase | 0 | 1 |
| M00525 | Lysine biosynthesis, acetyl-DAP pathway, aspartate => lysine | 0 | 2 |
| M00526 | Lysine biosynthesis, DAP dehydrogenase pathway, aspartate => lysine | 0 | 1 |
| M00532 | Photorespiration | 0 | 2 |
| M00545 | Trans-cinnamate degradation, trans-cinnamate => acetyl-CoA | 0 | 5 |
| M00549 | Nucleotide sugar biosynthesis, glucose => UDP-glucose | 0 | 1 |
| M00550 | Ascorbate degradation, ascorbate => D-xylulose-5P | 0 | 2 |
| M00554 | Nucleotide sugar biosynthesis, galactose => UDP-galactose | 0 | 1 |
| M00555 | Betaine biosynthesis, choline => betaine | 0 | 1 |
| M00568 | Catechol ortho-cleavage, catechol => 3-oxoadipate | 0 | 1 |
| M00569 | Catechol meta-cleavage, catechol => acetyl-CoA / 4-methylcatechol => propanoyl-CoA | 0 | 4 |
| M00577 | Biotin biosynthesis, BioW pathway, pimelate => pimeloyl-CoA => biotin | 0 | 1 |
| M00580 | Pentose phosphate pathway, archaea, fructose 6P => ribose 5P | 0 | 2 |
| M00609 | Cysteine biosynthesis, methionine => cysteine | 0 | 3 |
| M00625 | Methicillin resistance | 0 | 2 |
| M00627 | beta-Lactam resistance, Bla system | 0 | 3 |
| M00632 | Galactose degradation, Leloir pathway, galactose => alpha-D-glucose-1P | 0 | 1 |
| M00636 | Phthalate degradation, phthalate => protocatechuate | 0 | 1 |
| M00651 | Vancomycin resistance, D-Ala-D-Lac type | 0 | 2 |
| M00700 | Multidrug resistance, efflux pump AbcA | 0 | 1 |
| M00702 | Multidrug resistance, efflux pump NorB | 0 | 1 |
| M00704 | Tetracycline resistance, efflux pump Tet38 | 0 | 1 |
| M00705 | Multidrug resistance, efflux pump MepA | 0 | 1 |
| M00725 | Cationic antimicrobial peptide (CAMP) resistance, dltABCD operon | 0 | 4 |
| M00726 | Cationic antimicrobial peptide (CAMP) resistance, lysyl-phosphatidylglycerol (L-PG) synthase MprF | 0 | 1 |
| M00730 | Cationic antimicrobial peptide (CAMP) resistance, VraFG transporter | 0 | 2 |
| M00741 | Propanoyl-CoA metabolism, propanoyl-CoA => succinyl-CoA | 0 | 3 |
| M00781 | Nogalavinone/aklavinone biosynthesis, deoxynogalonate/deoxyaklanonate => nogalavinone/aklavinone | 0 | 1 |
| M00790 | Pyrrolnitrin biosynthesis, tryptophan => pyrrolnitrin | 0 | 1 |
| M00823 | Chlortetracycline biosynthesis, pretetramide => chlortetracycline | 0 | 1 |
| M00849 | C5 isoprenoid biosynthesis, mevalonate pathway, archaea | 0 | 4 |
| M00860 | Bacillus anthracis pathogenicity signature, polyglutamic acid capsule biosynthesis | 0 | 1 |
| M00861 | beta-Oxidation, peroxisome, VLCFA | 0 | 1 |
| M00868 | Heme biosynthesis, animals and fungi, glycine => heme | 0 | 1 |
| M00878 | Phenylacetate degradation, phenylaxetate => acetyl-CoA/succinyl-CoA | 0 | 2 |
| M00892 | UDP-N-acetyl-D-glucosamine biosynthesis, eukaryotes, glucose => UDP-GlcNAc | 0 | 1 |
| M00917 | Phytosterol biosynthesis, squalene 2,3-epoxide => campesterol/sitosterol | 0 | 1 |

**Fig. S1. Blueprint and corresponding photograph of 10 repeated ginseng monoculture**. The ginseng plants were cultured for 20 days, and the harvested roots were stored and organized for testing over a period of 1 day. Twenty-five ginseng plants were planted in each pot.

**Fig. S2. Disease index criteria used to assess the severity of root rot at the pot level.** Individual ginseng plants were categorized into five groups based on the extent of rot progression, and each group was assigned a score based on the median progression value. The overall score for each pot was determined by averaging the scores of individual plants, providing an effective and efficient representation of root rot severity.

**Fig. S3.** **Rarefaction curve of metagenome samples from monoculture model system of ginseng.** The upper graphs represent endosphere samples, while the lower graphs represent rhizosphere samples. The x-axis displays the number of reads obtained from the 16S rRNA V4 region. The y-axis represents the number of Amplicon Sequence Variants (ASVs) obtained after applying the divisive amplicon denoising algorithm. Chimera and non-bacterial reads were excluded. As the number of reads increases, the number of ASVs eventually converges to a steady value, indicating that the bacterial community in the sample has been adequately represented.

**Fig.** **S4. α diversity and relative abundance at the family level of the bacterial communities. A** The α diversity is described by three indices: Observed, Shannon, and Simpson, which represent the number of amplicon sequence variants (ASVs), the proportion of ASV abundance, and the evenness of the community, respectively **B** The top and bottom graphs represent the values for endosphere and rhizosphere, respectively. The bar graph in (B) displays the top 10 average abundance of the family level.

**Fig. S5. Correlation network analysis in endosphere.** The cumulative abundance of ASV at 95% in the endosphere was used for the SparCC network analysis. The correlation magnitude and positive or negative correlations are depicted by lines. The size of the nodes represents eigencentrality (*P* < 0.01, correlation magnitude > 0.5).

**Fig. S6.** $\boldsymbol{\beta}$**_RC_and** $\boldsymbol{\beta}$**NTIAnalysis.** $\beta$_RC_$\mathrm{and}\beta$NTI are two-tailed tests for comparing pairs in terms of phylogenetics. **A** a $\beta$_RC_ value less than -0.95 or greater than 0.95 indicates significant sharing or lack of sharing of community bacteria, respectively.$\mathbf{B} \beta$NTI value less than -2 or greater than 2 indicates significant community equivalence or difference.

**Fig. S7.** **Abundance prediction of amino acid biosynthesis pathway using PICRUSt2**. The figure illustrates the differences in abundance before and after turnover as determined by DESeq2 analysis. Yellow background panels indicate significant differences (*P_adj_* < 0.05, corrected using FDR method).

**Fig. S8.** **Predicted abundance of amino acid degradation pathways using PICRUSt2.** Differences were analyzed between pre- and post-turnover using DESeq2. Significantly different pathways (*P_adj_* < 0.05, FDR method) are highlighted with a yellow background.
